# A randomised, family-focused dietary intervention to evaluate the Atlantic diet: the GALIAT study protocol

**DOI:** 10.1186/s12889-016-3441-y

**Published:** 2016-08-18

**Authors:** Maria del Mar Calvo-Malvar, Rosaura Leis, Alfonso Javier Benítez-Estévez, Juan Sánchez-Castro, Francisco Gude

**Affiliations:** 1 Department of Laboratory Medicine, Clinic University Hospital of Santiago, Santiago de Compostela, Spain; 2 Unit of Paediatric Gastroenterology and Nutrition, Department of Paediatrics, Clinic University Hospital of Santiago, CiberObn, Santiago de Compostela, Spain; 3 A Estrada Primary Care Centre, Pontevedra, Spain; 4 Clinical Epidemiology Unit, Clinic University Hospital of Santiago, Santiago de Compostela, Spain

**Keywords:** Atlantic diet, Randomised controlled clinical trial, Family-focused diet intervention, Nutrition education, Study protocol, GALIAT study

## Abstract

**Background:**

The traditional diet of northwestern Spain and northern Portugal follows an ‘Atlantic diet’ pattern. Adherence to the Atlantic diet has been related to the good metabolic health and low coronary mortality recorded for these regions.

**Methods:**

The GALIAT (*Galicia Alimentación Atlántica* [Galicia Atlantic Diet]) study is a randomised, controlled, dietary intervention clinical trial designed to examine the effect of the Atlantic diet on the lipid profile, glucose metabolism, inflammation makers and adiposity of the general population. The trial involved 250 randomly selected families (715 adults and children over 3 years of age) from a town in Spain’s northwest, randomly allocated to follow either a control diet (C group) or the Atlantic diet (AD group) for a period of 6 months.

The families of the AD group received educational sessions on food, diet and gastronomy and were provided written supporting material with nutritional recommendations and recipes for the preparation of menus. They also attended cooking classes. Throughout the study period, these families were provided a range of foods (free of charge) that form part of the traditional Atlantic diet. The C group families took part in none of the above activities, nor were they provided with any food.

Lipid profile variables (primary variables), and anthropometric, inflammation marker and glucose metabolism status (secondary variables), were measured at baseline, three and six months.

**Discussion:**

The GALIAT study is the first clinical trial to examine the effects of the Atlantic diet on metabolic and cardiovascular health and adiposity. If the study hypothesis is confirmed, this dietary pattern could be included in strategies to promote health.

**Trial registration:**

ClinicalTrials.gov, NCT02391701 on March 18, 2015.

## Background

Spain has one of the lowest mortality rates for ischaemic heart disease in Europe [1]. However, wide variation in such mortality is seen across the country, with some towns in northern Spain returning figures up to 40 % lower than the mean [2]. Genetic factors may be involved, but diet and physical activity may also play an important role. In a population-based, case-control study in Porto, Portugal, Oliveira et al. [3] reported that adherence to the traditional Atlantic diet was associated with a lower probability of acute myocardial infarction. Adherence to this diet has also been associated with lower serum concentrations of inflammation markers, triglycerides and insulin, an improved insulin resistance index, and reduced systolic blood pressure [4].

Northern Portugal and Galicia (northwestern Spain) are geographically, climatically and culturally similar. The Atlantic diet they share is characterised by the abundant consumption of little-processed, local, fresh, seasonal foods, including fruit, vegetables, bread, cereals and pulses, along with fish and milk products. The consumption of meat (mainly beef and pork) and eggs is only moderate. The sauces used are low in calories but of high nutritional quality. Olive oil is used as a dressing and in cooking. Wine consumption (normally at mealtimes) is moderate. Food is commonly steamed, boiled, baked, grilled or stewed rather than fried. The dishes produced are not complicated but show care and originality in the combination of foods [5]. The Atlantic diet differs from the Mediterranean diet consumed in southern Spain by its greater intake of fish, milk, potatoes, fruit, vegetable and olive oil, and a greater intake of wine at the expense of beer [6]. The types of fruits and vegetables consumed are different too; the nutrients and functional components they provide are therefore also different. In addition, the preferred cooking techniques associated with the Atlantic diet modify the nutritional composition of foods less than frying.

No clinical trial has ever been performed to examine the effect of the Atlantic diet on health. The GALIAT (*Galicia Alimentación Atlántica* [Galicia Atlantic Diet]) study was designed to examine what effect it may have on the lipid profile, glucose metabolism, inflammation markers and adiposity of the general population, using the family as the intervention unit. The present work justifies the need for this study, describes its design, and explains how it was executed. The protocol adheres to the Consolidated Standards of Reporting Trials (CONSORT) recommendations for the reporting of non-pharmacological clinical trials [7].

## Methods

### Design

The GALIAT study is a randomised clinical trial with two parallel groups designed to examine the effect of a nutritional intervention involving the Atlantic diet. The study subjects - families from a town in northwestern Spain–were randomly assigned to one of two equally sized groups: a control group (C group) and an Atlantic diet group (AD group). “The family” formed the intervention unit, thus covering people from childhood to old age; it is within the family where nutritional and lifestyle habits are learned. Fieldwork was performed at a primary healthcare centre - an excellent strategic setting for preventing and intervening in risk factors affecting human health.

### Project coordination and location

The GALIAT study was designed by researchers at the *Hospital Clínico Universitario de Santiago de Compostela* (The Santiago de Compostela University Hospital) in Spain. Fieldwork, including the recruitment and follow-up of participating families was performed at the health centre in the rural town of A Estrada. A Estrada has a population of 22,362 and is some 27 km from the aforementioned hospital. This centre was chosen from among several candidates since its personnel have experience of observational studies. Twenty family doctors, three paediatricians and twenty nurses acted as collaborating researchers. In the three weeks before the study began, these persons were informed of the protocol and work procedures to follow. A further physician, nurse and four nutritionists were employed to lead the field study at the health centre. Their work involved interviewing the study subjects, the taking of anthropometric data, the measurement of blood pressure, obtaining biological samples, and verifying and transcribing data. The nutritionists were charged with performing the dietary intervention. All personnel involved in fieldwork received theoretical and practical instruction (one month before the study started) on how to normalise work procedures. This involved trial runs with volunteers subjected to all assessment processes. Daily meetings focused on the problems detected and their solution. Once the trial had begun, daily telephone calls and weekly physical meetings ensured its correct functioning. All fieldwork was performed in two doctor’s offices, plus a room used for the collection of biological samples, provided by the health centre.

### Sample size and power estimation

The sample size required to ensure a minimum predictive power of 80 % with a 0.05 type I error, assuming a 10 % drop-out rate, was determined to be 250 families. Calculations were performed using the Sample Size Shop’s GLIMMPSE 2.0 online tool for clustered data [8].

### Eligibility, recruitment, baseline screening and inclusion

**Fig. 1:**
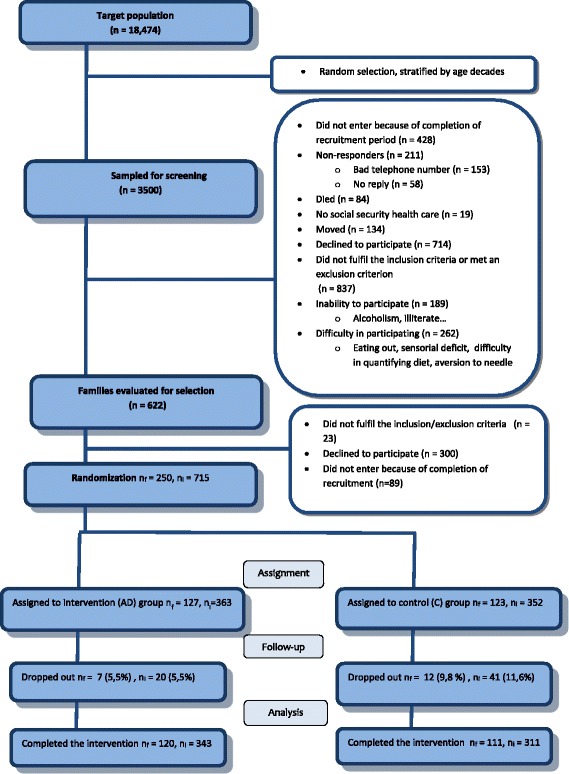
Flowchart showing numbers of subjects at each stage. n_f_, number of families, n_i_, number of individuals

To encourage the population to take part, the study was advertised in the press, on radio and television, and via posters hung at the health centre and the A Estrada town hall. The family doctors involved helped by answering questions about the project. Family selection was based on the random selection of index subjects from the National Health System records for the town. A random selection of 3500 individuals aged 18–85 years was made, stratified by decade. The chosen subjects were phoned in order to confirm their participation in the study. This provided a list of 662 subjects to whose homes letters explaining the project, and an invitation to take part, were sent. These subjects were again called by telephone to reconfirm their participation, to verify that those interested met the inclusion criteria, and to record the size of their families. Those who accepted the invitation to join the study were given a pre-assessment appointment at which time the project and its aim were verbally explained, an explanation provided regarding how subjects were selected, what participation would entail, and the rights of subjects defined. Documentation providing the details of the project, and consent forms for the whole family, were also distributed. Those who finally decided to take part were invited to return to the health centre, accompanied by their families, for baseline monitoring. The recruitment rate was 10–15 families per week. Figure 1 shows a flow diagram explaining the recruitment process and intervention phases.

### Inclusion and exclusion criteria

The inclusion criteria to be met by the index subject (male or female) of each family were: age 18-85 years, and to be part of a family (living together) of at least two members. The other members of the family (either sex) had to be aged 3–85 years.

The following were deemed reasons for excluding an index subject: alcoholism, undergoing lipid-lowering treatment, pregnancy, cardiovascular disease (ischaemic heart disease, heart failure, peripheral vascular disease, cerebrovascular disease), dementia, or having a predicted survival of less than one year. The exclusion criteria for the family members of the index subject were the same, except that those following lipid-lowering treatment were included. Finally, for a family to be incorporated into the study, at least two members had to meet all inclusion criteria but no exclusion criterion.

### Randomisation

The participating families were randomly assigned (1:1) by a person not associated with the study to either the C group or AD group via the use of computer-generated random numbers. This was performed at the baseline visit.

### Dietary intervention

#### The Atlantic diet

Table 1 shows the characteristics of the Atlantic diet.

**Table 1:** Food consumption recommendations

Daily	Servings/day
Bread, cereals, wholegrain cereals, rice, pasta and potatoes	6–8
Olive oil	3–4
Fruit	≥3
Vegetables	≥2
Milk products	3–4
Several Times Per Week	Servings/week
Fish and seafood	3–4
Lean meat	3–4
Eggs	3–4
Pulses	2–3
Nuts, preferably chestnuts, walnuts, almonds and hazelnuts^(a)^	4–6
Occasional	Servings/month
Fatty meat, cured sausage, margarine, butter	A few times per month
Sweets, pastries, cakes, candies, ice cream, etc.	A few times per month
Sugary drinks	A few times per month
Drink	Servings/day
Water	6–8

^(a)^ Only given to subjects ≥5 years of age for fear of choking, and made without salt

All participants in the AD group received the information, motivation and assistance necessary to modify their food habits in accordance with this diet. Since the palatability of the foods included is key if good adherence is to be achieved, a recipe book based on the use of local products was designed. Recipes were proposed by the research team, written out in detail by a chef, and the dishes calibrated by the team of nutritionists. Recommendations were drawn up so that the AD subjects’ daily and weekly food intakes would be well adjusted to both the Atlantic diet and consensus food recommendations [9–11]. This led to the publication of the recipe book “Platos y Menús Atlánticos” [12] (Atlantic Dishes and Menus; a pdf version is available from the authors), which includes all the recommendations, calibrated recipes, and information required for planning weekly menus. The nutritionists insisted upon the need for the subjects to use this book (which was distributed free to families in the AD group) during the study period. A day-long cooking course was also given to AD group members by the chef. A telephone number was supplied so that any doubts or problems could be discussed.

#### Trial protocol

The intervention period lasted 6 months. Table 2 describes the trial protocol, following the graphical layout of Perera et al. [13].

**Table 2:**
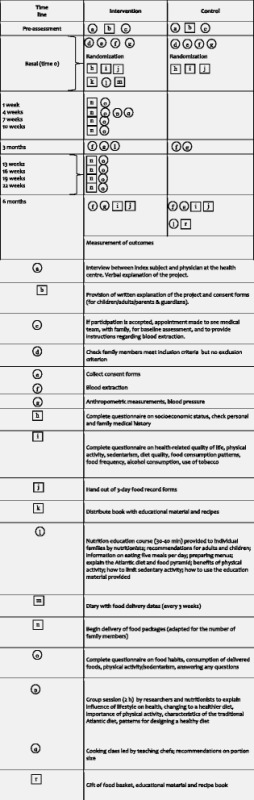
Graphical representation of the intervention

At the baseline visit all AD subjects received information on the Atlantic diet and how to follow it; the nutritionists’ messages were adapted to the clinical condition of each subject, his/her preferences, needs, beliefs and culture. Special care was taken with patients who were obese, had diabetes or who had high blood cholesterol; conflicts with recommendations made by subjects’ own doctors or nutritionists were avoided. All questionnaires (see below) were filled out in the presence of the nutritionists, except for 3-day food records which the subjects handed in completed. The latter were reviewed in the presence of the subjects in order to correct any errors or add missing information. At the 3 and 6 month visits, the nutritionists reminded the subjects about the Atlantic diet and the helped them complete their questionnaires.

Table 3 shows the foods provided to the families of the AD group. Wine was provided only to non-tee-total adults. Food packages were delivered every three weeks to the family home. The food included in the package was calculated for the total number of family members, even if not all were taking part in the study. At delivery, a form was filled in to verify that the previous lot of food had been consumed, and to note any problems.

**Table 3:** Quantity of food provided per Atlantic diet subject per week

Food	Quantity per week
Turnip greens (g)	250
Cabbage (g)	200
Mushrooms (g)	64
*Zaragallada* ^a^ (g)	175
Tomatoes (g)	277
Mirabelle plums (g)	140
Mussels (g)	57
Low fat cheese (g)	100
White wine (ml)	250
Red wine (ml)	250
Olive oil (ml)	330

g: drained weight

^a^ *zaragallada*: a tomato, green pepper and onion sauce used in Galicia to help fill samosa-like pies

### Data collection

Unless otherwise stated, data were collected at baseline, 3 and 6 months.

#### Demographic information and medical history collected

The following information was collected from all subjects.

1. *Sociodemographic characteristics -* place of birth, civil status, education, profession, whether in work, characteristics of the home.
2. *Personal and family medical history -* heart disease, diabetes, high blood pressure, cancer, hypercholesterolaemia, age at menarche and menopause.
3. *Medication -* lipid-lowering and blood pressure-lowering drugs.
4. *Health-related quality of life -* assessed using the Spanish v.2.0 of the Short Form 12 Health Survey (SF-12) questionnaire [14, 15]. Answers were interpreted with the use of reference values for Spanish populations [16].
5. *Tobacco and alcohol consumption.*

Table 4 summarises the information collected as well as the tests performed over the study period.

**Table 4:** Outcomes and time points for measurements

	Baseline	3 month	6 month
Questionnaires & Health Measurements			
Sociodemographic data	✓		
Health status: personal and family medical history	✓		
Quality of life, (SF-12v2 questionnaire)	✓		✓
Dietary habits, FFQ	✓		✓
3-day food record	✓		✓
Diet quality	✓		✓
Dietary patterns	✓		✓
IPAQ-short	✓		✓
Sedentary behaviour	✓		✓
Drinking habits (AUDIT-C questionnaire)	✓		✓
Use of tobacco	✓		✓
Anthropometry			
Body weight (kg)	✓	✓	✓
Height (cm)	✓	✓	✓
BMI (kg/m2)	✓	✓	✓
Waist circumference (cm)	✓		✓
Arm circumference (cm)	✓		✓
Thigh circumference (cm)	✓		✓
Biceps skinfold thickness	✓		✓
Triceps skinfold thickness	✓		✓
Subscapular skinfold thickness	✓		✓
Suprailiac skinfold thickness	✓		✓
Resting pulse and blood pressure	✓		✓
Biomarkers			
Total cholesterol	✓	✓	✓
HDL cholesterol	✓	✓	✓
LDL cholesterol	✓	✓	✓
Triglycerides	✓	✓	✓
Albumin	✓	✓	✓
Aspartate aminotransferase	✓	✓	✓
Alanine aminotransferase	✓	✓	✓
Gamma-glutamyl transferase	✓	✓	✓
Creatinine	✓	✓	✓
Urea	✓	✓	✓
Glucose	✓	✓	✓
HbA1c	✓	✓	✓
Insulin	✓		✓
Fructosamine	✓		✓
Leptin	✓		✓
C-reactive protein	✓		✓
Interleukin 6	✓		✓
Tumour necrosis factor α	✓		✓
25-OH cholecalciferol	✓	✓	✓
Thyrotropin	✓		✓
Haemogram	✓	✓	✓

### Monitoring food intake

#### Dietary quality

Dietary quality was assessed using the Krece Plus questionnaire [17] (Table 5). This assesses how well the actual diet of a subject compares to a healthy diet. It includes 16 items scored as either +1 or -1. The diet is then classified as either of high (≥9 points), medium (6–8 points), or low (≤5 points) nutritional value.

**Table 5:** Dietary quality index

Skips breakfast	−1
Has a dairy product for breakfast (yoghurt, milk, etc.)	+1
Has cereals or grains (bread, etc.) for breakfast	+1
Has commercially baked goods or pastries for breakfast	−1
Takes a fruit or fruit juice every day	+1
Has a second fruit every day	+1
Has a second dairy product every day	+1
Has fresh or cooked vegetables regularly once a day	+1
Has fresh or cooked vegetables more than once a day	+1
Consumes fish regularly (at least 2 – 3 times per week)	+1
Goes more than once a week to a fast-food (hamburger) restaurant	−1
Takes alcoholic drinks at least once a week	−1
Likes pulses and eats them more than once a week	+1
Takes sweets and candy several times every day	−1
Consumes pasta or rice almost every day (5 or more times per week)	+1
Uses olive oil at home	+1

#### Record of food intake: using food diaries

The study subjects completed a 3-day food record, including two weekdays and either a Saturday or Sunday, at baseline, 3 and 6 months (Table 3). Subjects were asked to provide information on brand names of foods consumed, preparation and cooking methods, to weigh all foods consumed when possible, and to use household measurements (spoonfuls, cupfuls) when not. All subjects were provided a weight/household measurement/“hand size” equivalency table for providing consumption information. All completed records were checked by the nutritionists.

At the moment of analysis, nutrient intake will be analysed using DIAL [18] software. This incorporates national and international tables on food composition, serving sizes and recipes. Dietary intake variables of interest include total energy (kcal) and intakes (%) of fat, protein, carbohydrate, fibre and micronutrients.

#### Food frequency questionnaire

Food intake over the last 4 weeks of the study was assessed using a semi-quantitative food frequency questionnaire (FFQ) which included 93 foods and drinks habitually consumed in Spain. This questionnaire was structured to include 13 food groups, 64 foodstuffs and 93 food items (since, e.g., milk can be either whole or skimmed), and was completed in the presence of a nutritionist, to record the mean daily, weekly or monthly consumption of each food, bearing in mind serving size. Supporting material was provided so that subjects could identify serving sizes. For most items a standard portion was the recording unit (e.g., a 250 ml glass of milk, a carton of yoghurt, a piece of fruit, a slice of bread). A portion of boiled vegetables was regarded as 200 g, a portion of lettuce as 100 g, a portion of sugary soda drink as a 330 ml canful, and a portion of wine as a 100 ml glassful. Frequency of consumption was expressed on a nine-point ordinal scale (never or hardly ever, 1–3 times per week, 2–4 times per week, 5–6 times per week, once per day, 2–3 times per day, 4–6 times per day, over 6 times per day).

#### Assessment of physical activity and sedentary behaviour

Subjects completed the International Physical Activity Questionnaire (short format) [19], from which the metabolic equivalents and hours per week spent in vigorous and moderate activities, and in walking (as described by Craig et al. [20]) were calculated. This classified subjects as ‘inactive’, ‘minimally active’, and ‘HEPA active’ (health enhancing physical activity; a high activity category).

For subjects under 18 years of age, the enKid rapid questionnaire (the Krece Plus Short Physical Activity Test) [17] was used. Its questions relate to time spent getting to and from school, and time spent in sporting activity at school and outside school. The time spent in inactivity and in light, moderate and vigorous activity can then be determined. This test classifies a person’s lifestyle based on the mean number of hours per day spent watching television or playing videogames, and the number of hours spent in sport (outside of school) per week. Lifestyles are classified as poor, regular or good.

For subjects under 18 years of age, the HELENA Sedentary Behaviour Questionnaire [21] was used to assess the amount of time per week spent in 11 types of sedentary behaviour. For the adult subjects an in-house adapted version was used. The questions assess the number of hours a subject spends working, travelling and at home, taking particular note of how much time is spent using a computer at home. For subjects under 18 years of age an unmodified version of the questionnaire was used.

#### Anthropometric measurements

All measurements were made in triplicate. Body weight and height were recorded by standard methods using a SECA 813 digital balance and a SECA 213 stadiometer respectively, with the subjects in underwear and barefoot. Body weight was measured to the nearest 0.1 kg and body height to the nearest 0.1 cm. Body mass index (BMI) was calculated as body weight (kg) divided by height (m) squared. The BMI of subjects under 18 years of age was standardised using WHO reference data [22].

Waist and hip circumferences were measured using a SECA 201 flexible, non-elastic tape. Waist circumference was measured at the narrowest point between the bottom rib and the top of the iliac crest; hip circumference was measured at the point of greatest prominence of the gluteal muscles. Skinfold thicknesses (see Table 4) were measured using a HOLTAIN Taner Whitehouse caliper. Blood pressure was recorded using an OMRON M3 automatic sphygmomanometer after subjects had been seated for 5 min.

#### Biomarkers

All laboratory analyses were performed at the Santiago University Hospital. Blood was extracted under standard conditions between 08.00 and 10.00 h following a 10–14 h fast; extractions began after the subjects had been seated for a minimum 5 min and had shown venous stasis for under 2 min.

Glucose, creatinine, urea, albumin, aspartate aminotransferase, gamma-glutamyl transferase, thyrotropin, total cholesterol, HDL cholesterol and triglycerides were measured using an Advia 2400 Clinical Chemistry System (Siemens Healthcare Diagnostics). LDL cholesterol was estimated using the Friedewald formula [23], except when triglycerides were over 400 mg/dl, in which case an enzymatic method was employed with determinations made using the same autoanalyser. Fructosamine was determined using the Genzyme GlyPro enzymatic method adapted for the above autoanalyser. High sensitivity C-reactive protein, insulin and serum interleukin-6 was determined using an immunometric chemoluminiscence method, employing an Immulite 2000 Immunoassay System. Tumour necrosis factor α was determined in serum using an Immulite 1000 Immunoassay System. Serum thyrotropin was determined by chemoluminiscent immunoassay, and serum 25-OH cholecalciferol by competitive immunoassay, in both cases employing an Advia Centaur Analyzer (Siemens Healthcare Diagnostics). HBA1c was determined via high resolution liquid chromatography using a Menarini Diagnostics HA-8160 Analyzer; all results were referenced to DCCT units [24]. Leptin was determined by sandwich ELISA using a DRG Diagnostics kit (Marburg, Germany). All biochemical determinations were made on the day of blood extraction. Aliquots of samples were preserved at -80 °C for later determination of hormones, adipocines, and for possible future genetic studies.

### Statistical analysis

At the time of analysis, descriptive statistics will be calculated for all covariates at 0, 3 and 6 months, and according to trial arm. Lipid and inflammatory markers will be analysed on an intent to treat basis; thus, analyses will include every subject randomised regardless of final protocol adherence, study completion, or missing data. Where follow-up values are missing, multiple imputation will be performed [25]. Linear mixed models adjusted for baseline differences will be used to examine differences between the C and AD groups with the intervention condition deemed a fixed effect and clusters (family) as a random effect. Principal components analysis will be used to identify dietary patterns. All statistical analyses will be carried out using R software, employing the NLME package for fitting linear mixed-effects models, the MICE package for multiple imputation, and the NSPRCOMP package for performing principal components analysis (all freely available at http://cran.r-project.org [26]).

## Discussion

This work presents a clinical trial protocol involving a dietary intervention that focuses on: (i) the use of an entire dietary pattern rather than a reduced number of foods, (ii) the family as the intervention unit, (iii) and a representative sample of the general population.

In a population-based case-control study, Oliveira et al. [3] reported an inverse association between adherence to the Atlantic diet and non-fatal myocardial infarction. Later, Guallar-Castillón et al. [4], in a cross-sectional study on a large cohort of Spanish adults, reported adherence to this diet to be related to lower blood levels of coronary risk biochemical markers. The GALIAT study is the first clinical trial designed to assess the effects of the Atlantic diet on metabolic and cardiovascular health and adiposity. Further, the study involves a wide range of diet-related factors (dietary habits, food consumption), lifestyles (physical activity, use of tobacco, sedentary habits, socioeconomic characteristics, health characteristics and culture), and examines a wide range of biomarkers. The delivery of foods to the subjects’ homes, the educational sessions, the cooking lessons, and the support material provided should help minimise drop-out and facilitate adherence.

Dietary intervention studies have many limitations that present challenges to trial designers [27]. A limitation of the present study could be contamination bias; the study was undertaken in one town and received notable coverage by local and national news outlets. This could mean that some people of the control group might have adopted a more Atlantic-type diet during the study period, which could skew any assessment of the intervention towards an acceptance of the null hypothesis. Further, subjects can always complete questionnaires with what they imagine to be more desirable answers and thus receive approbation (obsequiousness bias). In addition, answers regarding food intakes and lifestyles are always somewhat subjective [27, 28]. Moreover, since this is an open label trial, observer/interviewer bias is possible, i.e., the interviewers could involuntarily help the subjects when answering questionnaires, thus introducing bias. Finally, the duration of the trial - 6 months – may be insufficient to affirm long term changes in adiposity.

In conclusion, the aim of this work is to examine the scientific evidence that might justify the promotion of the Atlantic diet as a healthy choice, and thus allow it to be incorporated into preventive family health strategies in line with the cultural and gastronomic heritage of Europe’s Atlantic regions.

## Abbreviations

BMI, body mass index; DCCT, diabetes control and complications trial; FFQ, food frequency questionnaire; MET, metabolic equivalent of task
